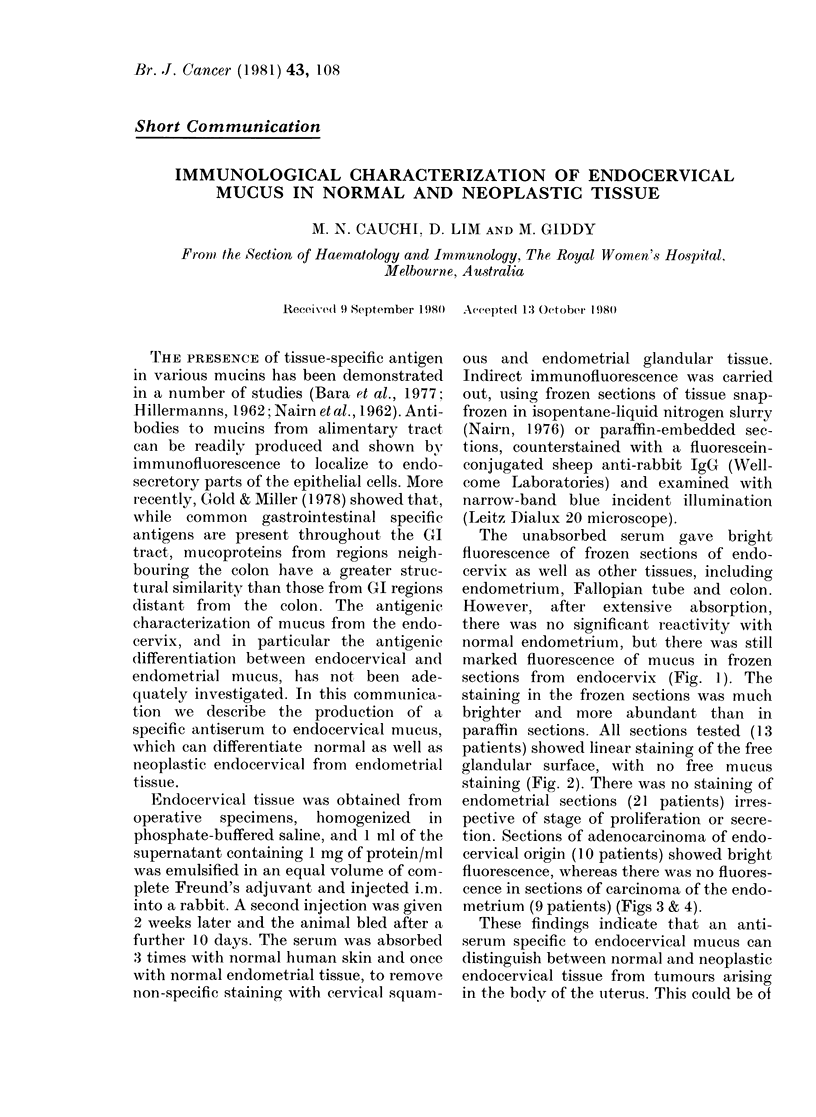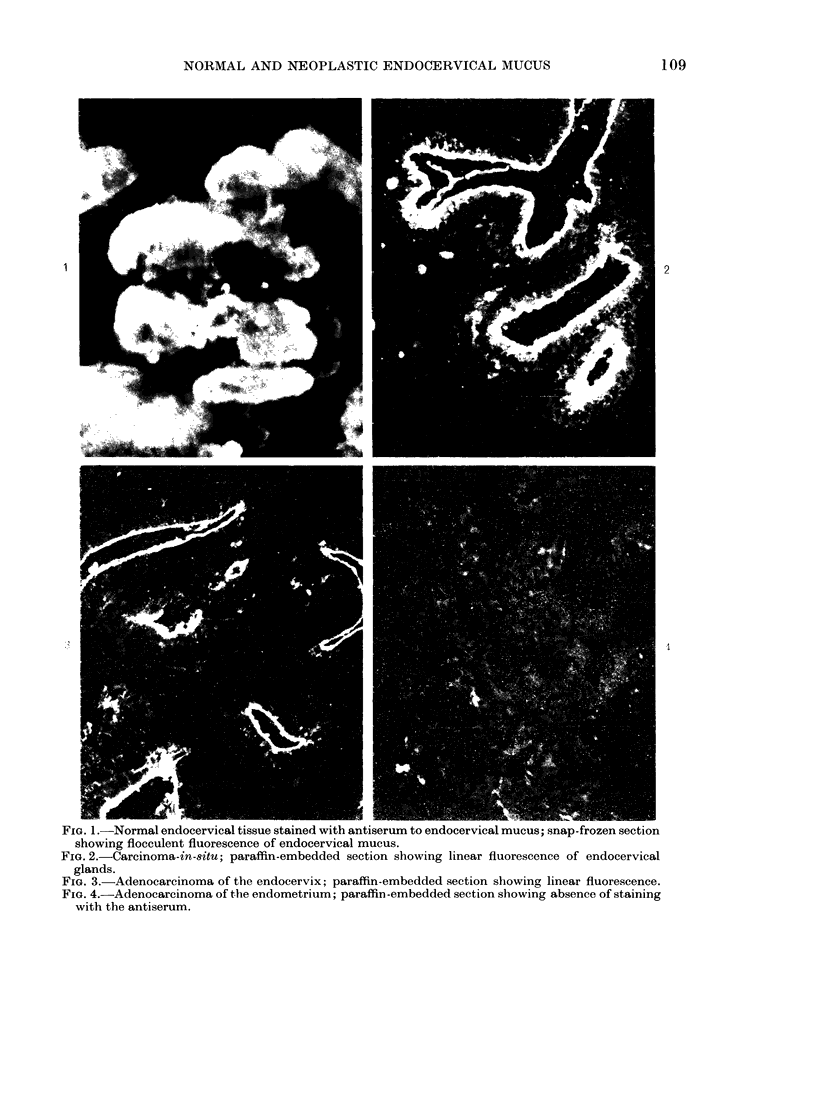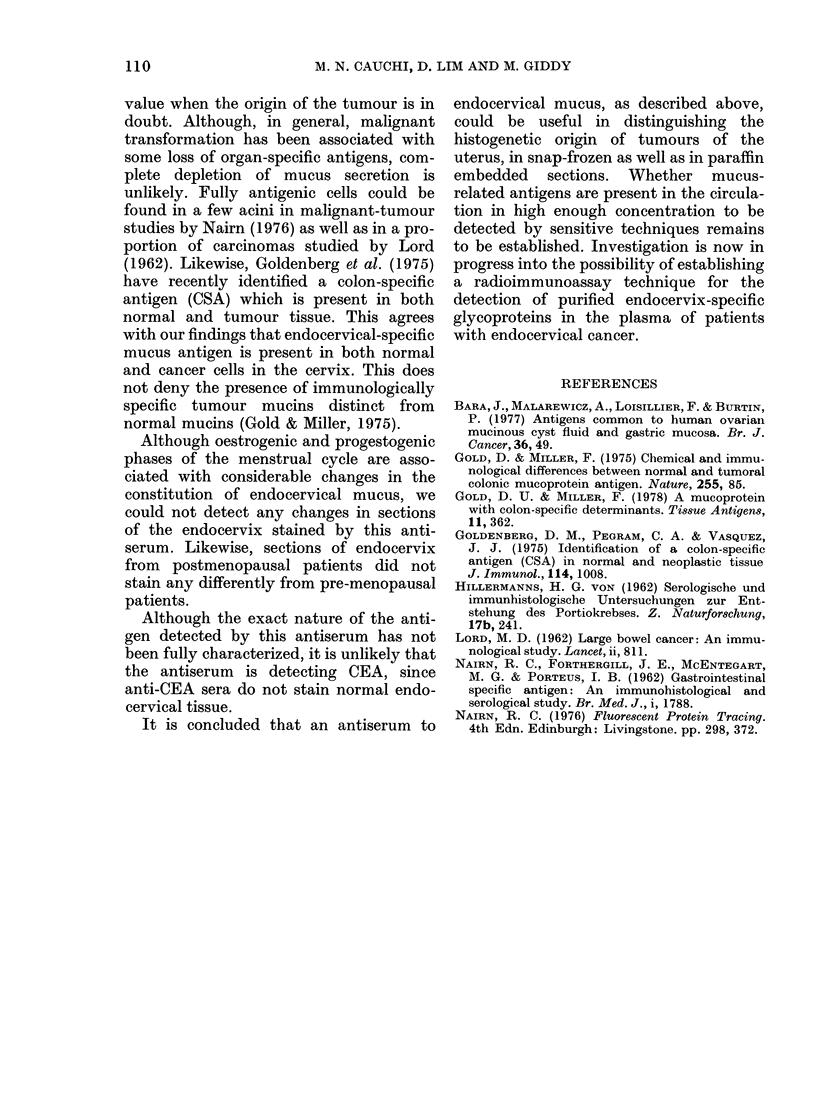# Immunological characterization of endocervical mucus in normal and neoplastic tissue.

**DOI:** 10.1038/bjc.1981.16

**Published:** 1981-01

**Authors:** M. N. Cauchi, D. Lim, M. Giddy

## Abstract

**Images:**


*Br. J. Cancer* (1981) **43**, 108

***Short Communication***

### IMMUNOLOGICAL CHARACTERIZATION OF ENDOCERVICAL MUCUS IN NORMAL AND NEOPLASTIC TISSUE

M. N. CAUCHI, D. LIM AND M. GIDDY

*From the Section of Haematology and Immunology, The Royal Women's Hospital, Melbourne, Australia*

Received 9 September 1980 Accepted 13 October 1980

THE PRESENCE of tissue-specific antigen in various mucins has been demonstrated in a number of studies (Bara *et al.*, 1977; Hillermanns, 1962; Nairn *et al.*, 1962). Antibodies to mucins from alimentary tract can be readily produced and shown by immunofluorescence to localize to endosecretory parts of the epithelial cells. More recently, Gold & Miller (1978) showed that, while common gastrointestinal specific antigens are present throughout the GI tract, mucoproteins from regions neighbouring the colon have a greater structural similarity than those from GI regions distant from the colon. The antigenic characterization of mucus from the endocervix, and in particular the antigenic differentiation between endocervical and endometrial mucus, has not been adequately investigated. In this communication we describe the production of a specific antiserum to endocervical mucus, which can differentiate normal as well as neoplastic endocervical from endometrial tissue.

Endocervical tissue was obtained from operative specimens, homogenized in phosphate-buffered saline, and 1 ml of the supernatant containing 1 mg of protein/ml was emulsified in an equal volume of complete Freund's adjuvant and injected i.m. into a rabbit. A second injection was given 2 weeks later and the animal bled after a further 10 days. The serum was absorbed 3 times with normal human skin and once with normal endometrial tissue, to remove non-specific staining with cervical squamous and endometrial glandular tissue. Indirect immunofluorescence was carried out, using frozen sections of tissue snap-frozen in isopentane-liquid nitrogen slurry (Nairn, 1976) or paraffin-embedded sections, counterstained with a fluorescein-conjugated sheep anti-rabbit IgG (Wellcome Laboratories) and examined with narrow-band blue incident illumination (Leitz Dialux 20 microscope).

The unabsorbed serum gave bright fluorescence of frozen sections of endocervix as well as other tissues, including endometrium, Fallopian tube and colon. However, after extensive absorption, there was no significant reactivity with normal endometrium, but there was still marked fluorescence of mucus in frozen sections from endocervix (Fig. 1). The staining in the frozen sections was much brighter and more abundant than in paraffin sections. All sections tested (13 patients) showed linear staining of the free glandular surface, with no free mucus staining (Fig. 2). There was no staining of endometrial sections (21 patients) irrespective of stage of proliferation or secretion. Sections of adenocarcinoma of endocervical origin (10 patients) showed bright fluorescence, whereas there was no fluorescence in sections of carcinoma of the endometrium (9 patients) (Figs 3 & 4).

These findings indicate that an antiserum specific to endocervical mucus can distinguish between normal and neoplastic endocervical tissue from tumours arising in the body of the uterus. This could be of

FIG. 1.—Normal endocervical tissue stained with antiserum to endocervical mucus; snap-frozen section showing flocculent fluorescence of endocervical mucus.

FIG. 2.—Carcinoma-*in-situ*; paraffin-embedded section showing linear fluorescence of endocervical glands.

FIG. 3.—Adenocarcinoma of the endocervix; paraffin-embedded section showing linear fluorescence.

FIG. 4.—Adenocarcinoma of the endometrium; paraffin-embedded section showing absence of staining with the antiserum.

value when the origin of the tumour is in doubt. Although, in general, malignant transformation has been associated with some loss of organ-specific antigens, complete depletion of mucus secretion is unlikely. Fully antigenic cells could be found in a few acini in malignant-tumour studies by Nairn (1976) as well as in a proportion of carcinomas studied by Lord (1962). Likewise, Goldenberg *et al.* (1975) have recently identified a colon-specific antigen (CSA) which is present in both normal and tumour tissue. This agrees with our findings that endocervical-specific mucus antigen is present in both normal and cancer cells in the cervix. This does not deny the presence of immunologically specific tumour mucins distinct from normal mucins (Gold & Miller, 1975).

Although oestrogenic and progestogenic phases of the menstrual cycle are associated with considerable changes in the constitution of endocervical mucus, we could not detect any changes in sections of the endocervix stained by this antiserum. Likewise, sections of endocervix from postmenopausal patients did not stain any differently from pre-menopausal patients.

Although the exact nature of the antigen detected by this antiserum has not been fully characterized, it is unlikely that the antiserum is detecting CEA, since anti-CEA sera do not stain normal endocervical tissue.

It is concluded that an antiserum to endocervical mucus, as described above, could be useful in distinguishing the histogenetic origin of tumours of the uterus, in snap-frozen as well as in paraffin embedded sections. Whether mucus-related antigens are present in the circulation in high enough concentration to be detected by sensitive techniques remains to be established. Investigation is now in progress into the possibility of establishing a radioimmunoassay technique for the detection of purified endocervix-specific glycoproteins in the plasma of patients with endocervical cancer.

### REFERENCES

BARA, J., MALAREWICZ, A., LOISILLIER, F. & BURTIN, P. (1977) Antigens common to human ovarian mucinous cyst fluid and gastric mucosa. *Br. J. Cancer*, **36**, 49.

GOLD, D. & MILLER, F. (1975) Chemical and immunological differences between normal and tumoral colonic mucoprotein antigen. *Nature*, **255**, 85.

GOLD, D. U. & MILLER, F. (1978) A mucoprotein with colon-specific determinants. *Tissue Antigens*, **11**, 362.

GOLDENBERG, D. M., PEGRAM, C. A. & VASQUEZ, J. J. (1975) Identification of a colon-specific antigen (CSA) in normal and neoplastic tissue *J. Immunol.*, **114**, 1008.

HILLERMANNS, H. G. VON (1962) Serologische und immunhistologische Untersuchungen zur Entstehung des Portiokrebses. *Z. Naturforschung*, **17b**, 241.

LORD, M. D. (1962) Large bowel cancer: An immunological study. *Lancet*, ii, 811.

NAIRN, R. C., FORTHERGILL, J. E., MCENTEGART, M. G. & PORTEUS, I. B. (1962) Gastrointestinal specific antigen: An immunohistological and serological study. *Br. Med. J.*, i, 1788.

NAIRN, R. C. (1976) *Fluorescent Protein Tracing.* 4th Edn. Edinburgh: Livingstone. pp. 298, 372.